# Supplementary material for: A Real-World Retrospective Analysis of the Management of Advanced Urothelial Carcinoma in Canada
Source: Curr Oncol. 2024 Jan 25;31(2):704–22. doi: 10.3390/curroncol31020052 (PMC10887988; doi:10.3390/curroncol31020052)
Supplement: Supplementary file 1 [file curroncol-31-00052-s001.zip › curroncol-2780526-supplementary.pdf]

Table S1. Medical chart review questionnaire

About My Practice

1. **Please indicate your specialty:**
   - ❑ Medical oncologist
   - ❑ Hematologist oncologist
   - ❑ Uro-oncologist
   - ❑ Other (please specify):
2. **Please indicate the province where your practice is based:**
   - ❑ Alberta
   - ❑ British Columbia
   - ❑ Manitoba
   - ❑ New Brunswick
   - ❑ Newfoundland and Labrador
   - ❑ Nova Scotia
   - ❑ Ontario
   - ❑ Prince Edward Island
   - ❑ Quebec
   - ❑ Saskatchewan
   - ❑ Yukon
   - ❑ Northwest Territories
   - ❑ Nunavut
3. **What is the primary setting of your practice?**
   - ❑ Academic- / University-affiliated hospital
   - ❑ Community-based hospital / clinic / practice
   - ❑ Other (please specify):
4. **How many years have you been in practice?**
   - ❑ 0-10 years
   - ❑ 10-20 years
   - ❑ 20-30 years
   - ❑ 30+ years
5. **How many new patients with locally advanced unresectable or metastatic urothelial carcinoma did you care for in the last month?**
   - ❑ 0
   - ❑ 1-2
   - ❑ 3-5
   - ❑ 5-10
   - ❑ 10+

Patient Information

6. **Sex:**
   - ❑ Male
   - ❑ Female
7. **Age (years): ___________**
8. **When was the patient diagnosed with locally advanced unresectable or metastatic urothelial carcinoma?**
   [Drop down menu for month and year of diagnosis]

Medical History

9. Does this patient have newly diagnosed locally advanced unresectable or metastatic urothelial carcinoma (aUC) or has the patient progressed from an earlier stage of urothelial carcinoma?

- ❑ Newly diagnosed aUC
- ❑ Disease progression from early-stage disease

9.1. *[If Newly diagnosed aUC is selected in question 9]*

What symptoms led to suspicion of aUC? (Select all that apply.)

- ❑ Painless gross hematuria
- ❑ Irritative bladder symptoms (e.g., dysuria, urgency, frequency of urination)
- ❑ Obstructive voiding symptoms
- ❑ Pelvic or bony pain
- ❑ Lower-extremity edema (from iliac vessel compression)
- ❑ Flank pain (from ureteral obstruction)
- ❑ Palpable mass on physical examination
- ❑ Constitutional symptoms (e.g., fatigue, weight loss, anorexia)
- ❑ Other (please specify): ____________

9.2. *[If Disease progression from early-stage disease is selected in question 9]*

What was the primary tumor (T) stage when the patient was first diagnosed with localized disease?

- ❑ Ta, papillary tumor confined to the epithelium
- ❑ T1, tumor invasion into the lamina propria
- ❑ T2, tumor invasion into the muscularis propria

  [If selected:]
  - ❑ T2a, superficial muscularis propria
  - ❑ T2b, deep muscularis propria
- ❑ T3, tumor involvement of the perivesical fat

  [If selected:]
  - ❑ T3a, microscopic invasion
  - ❑ T3b, macroscopic invasion
- ❑ T4, tumor involvement of adjacent organs

  [If selected:]
  - ❑ T4a, invasion of prostatic stroma, seminal vesicles, uterus, or vagina
  - ❑ T4b, invasion of pelvic or abdominal wall

9.3. *[If Disease progression from early-stage disease is selected in question 9]*

What was the node (N) stage when the patient was first diagnosed with localized disease?

- ❑ N0, no regional lymph node metastasis
- ❑ N1, metastasis in a single lymph node in true pelvis
- ❑ N2, metastasis in multiple regional lymph nodes in true pelvis
- ❑ N3, metastasis in common iliac lymph node(s)

9.4. *[If Disease progression from early-stage disease is selected in question 9]*

Did the patient undergo a cystectomy?

- ❑ Yes

  [If selected, ask:] **Please indicate the time of the cystectomy:** [Dropdown menu to select year and month]
- ❑ No

10. What is the patient's metastatic disease status? (Select all that apply.)

- ❑ No distant metastasis (locally advanced unresectable)
- ❑ Lymph node metastases beyond the common iliacs
- ❑ Bone metastases
- ❑ Lung metastases
- ❑ Liver metastases
- ❑ Other (please specify): ____________________

11. What steps were involved in the initial evaluation of the advanced disease? (Select all that apply.)

- ❑ History and physical examination
- ❑ Examination under anesthesia (bimanual)
- ❑ Urine cytology
- ❑ Cystoscopy (standard white light)
- ❑ Enhanced cystoscopy (e.g., fluorescence)
- ❑ Biopsy
- ❑ Transurethral resection of bladder tumor (TURBT)
- ❑ Urinary tract imaging

11.1. [If selected:]

- ❑ Computed tomography (CT) urogram
- ❑ Magnetic resonance imaging (MRI) urogram
- ❑ Renal ultrasound
- ❑ Intravenous or retrograde pyelogram (IVP)

- ❑ Metastatic workup (imaging)

11.2. [If selected:]

- ❑ Chest imaging (CT scan or chest radiograph)
- ❑ Bone scan (radionucleotide scan)
- ❑ 18F-fluorodeoxyglucose (FDG)-Positron emission tomography (PET)/CT

- ❑ Estimate GFR to assess eligibility for cisplatin

12. What is your creatinine clearance threshold when assessing eligibility for cisplatin?

- ❑ 60 mL/min
- ❑ 55 mL/min
- ❑ 50 mL/min
- ❑ 45 mL/min
- ❑ Other (please specify): __________

13. What method do you use to assess kidney function?

- ❑ Measured creatinine clearance
- ❑ Estimated creatinine clearance using Cockroft-Gault variant
- ❑ Measured GFR
- ❑ Estimated GFR using the MDRD equation
- ❑ Estimated GFR using the CKD-EPI (2012) equation
- ❑ Estimated GFR using the CKD-EPI (2021) equation

14. What molecular markers have you tested, if any?

- ❑ Programmed death-ligand 1 (PD-L1) expression

14.1. [If selected:]

- ❑ Positive
- ❑ Negative
- ❑ Results pending

- ❑ Fibroblast growth factor receptor (FGFR) 3 genetic alteration

14.2. [If selected:]

- ❑ Positive
- ❑ Negative
- ❑ Results pending

- ❑ I do not routinely perform molecular diagnostics for bladder cancer

15. Does the patient have any comorbidities?

- ❑ Yes

15.1. *[If selected, ask]:* **What comorbidities does your patient have? (Select all that apply.)**

- ❑ Chronic kidney disease
- ❑ Dyslipidemia
- ❑ Diabetes
- ❑ Hypertension
- ❑ Obesity / overweight
- ❑ Atherosclerotic cardiovascular disease

- ❑ Heart failure
- ❑ Gastrointestinal disease
- ❑ Major psychiatric disorder
- ❑ Other (please specify): ________

❑ No

16. Does the patient have a history of smoking?

❑ Yes

16.1. [If selected:]

- ❑ Current
- ❑ Former

❑ No

Management

17. What are your priority treatment goals for this patient? (Select the top 3.)

- ❑ Improve survival
- ❑ Delay progression-free survival
- ❑ Improve response rates
- ❑ Reduce burden of disease
- ❑ Improve quality of life
- ❑ Symptom management
- ❑ Other (please specify): __________

18. Is the patient currently on first-line platinum-based chemotherapy?

❑ Yes

18.1. *[If Yes selected, ask:]* **What first-line chemotherapy is the patient receiving?**

- ❑ Gemcitabine and cisplatin (GC)
- ❑ Gemcitabine and carboplatin
- ❑ Methotrexate, vinblastine, doxorubicin, and cisplatin (MVAC)
- ❑ Dose-dense MVAC (ddMVAC)
- ❑ Other (please specify): _________

❑ No

18.2. *[If No selected, ask]:* **What first-line chemotherapy did your patient receive?**

- ❑ Gemcitabine and cisplatin (GC)
- ❑ Gemcitabine and carboplatin
- ❑ Methotrexate, vinblastine, doxorubicin, and cisplatin (MVAC)
- ❑ Dose-dense MVAC (ddMVAC)
- ❑ Other (please specify): _________

18.3. *[If No selected, also ask]:* **What was your patient's response to first-line chemotherapy?**

- ❑ Complete response
- ❑ Partial response
- ❑ Stable disease
- ❑ Disease progression

19. Why did you select this first-line therapy for your patient? (Select the top 3 reasons.)

- ❑ Most efficacious option for this patient
- ❑ Favourable safety profile
- ❑ Associated with good adherence
- ❑ Tolerable
- ❑ Patient preference
- ❑ Eligible for cisplatin according to Galsky criteria
- ❑ NOT eligible for cisplatin according to Galsky criteria

19.1. *[If selected:]* **Indicate which criteria:**

- ❑ ECOG performance status ≥ 2
    - ❑ Creatinine clearance < 60 mL/min
    - ❑ Grade of audiometric hearing loss ≥ Grade 2 (CT-CAE)
    - ❑ Grade of peripheral neuropathy ≥ Grade 2 (CT-CAE)
    - ❑ New York Heart Association heart failure Grade III/IV
- ❑ Patient is fit
- ❑ Patient is frail (e.g., multiple comorbidities, advanced age)
- ❑ Access
- ❑ Only therapy indicated for this patient
- ❑ My "go-to" for most first-line patients
- ❑ Other (please specify): ________

If patient is currently on first-line platinum-based chemotherapy (Yes to question 18), ask:

20. Are you planning maintenance therapy with avelumab after completing chemotherapy?

- ❑ Yes

20.1. *[If selected, ask:]* **How many weeks after completing chemotherapy will you start maintenance therapy?**

- ❑ < 4 weeks
- ❑ 4-6 weeks
- ❑ 6-8 weeks
- ❑ 8-10 weeks
- ❑ >10 weeks

- ❑ No

20.2. *[If selected, ask:]* **Please indicate why. (Select all that apply.)**

- ❑ Patient preference
- ❑ Concerns about patient's ability to tolerate maintenance therapy
- ❑ Minimal residual disease following platinum-based chemotherapy
- ❑ Partial response following platinum-based chemotherapy
- ❑ Stable disease following platinum-based chemotherapy
- ❑ Significant immunosuppression
- ❑ Concomitant immunosuppressive therapy
- ❑ Poor performance status
- ❑ Elderly patient
- ❑ Pre-existing medical condition
- ❑ Other (please specify): ________

If patient is NOT currently on first-line platinum-based chemotherapy (No to question 18) ask question 21:

21. Why is the patient no longer on first-line chemotherapy?

- ❑ Currently on maintenance therapy or starting maintenance therapy in the coming weeks
- ❑ Surveillance
- ❑ Disease progression, on second-line therapy or beyond

If patient is currently on maintenance therapy or starting therapy soon (see question 21), ask questions 21 to 25:

22. In addition to you (the treating physician), who is involved in patient care? (Select all that apply.)

- ❑ Infusion nurse
- ❑ Clinic nurse
- ❑ Pharmacist
- ❑ Other (please specify): _______

22.1. *[If any of the above are selected, ask:]* **What is their role? (Select all that apply.)**

- ❑ Counsel after initial diagnosis
- ❑ Ongoing counsel during treatment
- ❑ Monitor patient for signs and symptoms of infusion-related reaction

- ❑ Manage infusion-related reaction
- ❑ Monitor patient for treatment-emergent adverse events
- ❑ Manage supportive treatment(s)
- ❑ Adjust concomitant medications

23. Has your patient experienced any adverse event(s) that was(were) difficult or time-consuming to manage?

- ❑ No
- ❑ Yes

23.1. *[If selected:]* **What was the adverse event? (Select all that apply.)**

- ❑ Anemia
- ❑ Arthralgia
- ❑ Asthenia
- ❑ Back pain
- ❑ Constipation
- ❑ Cough
- ❑ Decreased appetite
- ❑ Diarrhea
- ❑ Fatigue
- ❑ Hematuria
- ❑ Immune-mediated adverse reaction

[If selected:] **Please specify:**

- ❑ Endocrinopathy
- ❑ Hepatitis
- ❑ Nephritis and renal dysfunction
- ❑ Pneumonitis
- ❑ **Other immune-mediated adverse reaction (please specify): ______**

- ❑ Infusion-related reaction
- ❑ Nausea
- ❑ Pruritus
- ❑ Pyrexia
- ❑ Rash
- ❑ Urinary tract infection
- ❑ Vomiting
- ❑ Other (please specify): _______

24. How was the safety event managed? (Select all that apply.)

- ❑ No intervention required
- ❑ Managed with supportive treatment(s) (e.g., corticosteroids)
- ❑ Withheld dose, no supportive treatment(s) required
- ❑ Withheld dose, managed with supportive treatment(s)
- ❑ Patient was referred to another specialist for further assessment
- ❑ Permanent discontinuation of maintenance therapy

25. What second-line therapy are you planning for this patient in case of disease progression?

- ❑ Reinduction with platinum-based chemotherapy
- ❑ Gemcitabine
- ❑ Paclitaxel
- ❑ Erdafitinib
- ❑ Enfortumab vedotin
- ❑ Pembrolizumab or immunotherapy alternative (avelumab, durvalumab)
- ❑ Clinical trial
- ❑ Too early to tell/ have not planned that far ahead
- ❑ Other (please specify): __________

If patient is currently under surveillance (see question 21), ask questions 26 and 27:

26. Why is the patient <u>not</u> on maintenance therapy? (Select all that apply.)

- ❑ Patient preference
- ❑ Concerns about patient's ability to tolerate maintenance therapy
- ❑ Minimal residual disease following platinum-based chemotherapy
- ❑ Partial response following platinum-based chemotherapy
- ❑ Stable disease following platinum-based chemotherapy
- ❑ Significant immunosuppression
- ❑ Concomitant immunosuppressive therapy
- ❑ Poor performance status
- ❑ Elderly patient
- ❑ Pre-existing medical condition
- ❑ Other (please specify): ________

27. What second-line therapy are you planning for this patient in case of disease progression?

- ❑ Reinduction with platinum-based chemotherapy
- ❑ Gemcitabine
- ❑ Paclitaxel
- ❑ Erdafitinib
- ❑ Enfortumab vedotin
- ❑ Pembrolizumab or immunotherapy alternative (avelumab, durvalumab)
- ❑ Clinical trial
- ❑ Too early to tell/ have not planned that far ahead
- ❑ Other (please specify): __________

If patient had disease progression and is on second-line therapy or beyond (see question 21), ask questions 28 to 32:

28. How long was your patient on maintenance therapy before discontinuing therapy?

[drop down menu to select number of months, ranging from 0 to 36]

29. Why was maintenance therapy discontinued?

- ❑ Disease progression
- ❑ Adverse event
- ❑ Patient can no longer tolerate therapy
- ❑ Patient preference
- ❑ Other (please specify): ________

30. Did you ever have to withhold maintenance therapy?

- ❑ Yes

30.1. *[If selected, ask:]* **What was the reason for the hold?**

- ❑ Adverse event
- ❑ Drug holiday
- ❑ Other (please specify): _______

- ❑ No

31. What second-line therapy was chosen for this patient?

- ❑ Reinduction with platinum-based chemotherapy
- ❑ Gemcitabine
- ❑ Paclitaxel
- ❑ Erdafitinib
- ❑ Enfortumab-vedotin
- ❑ Pembrolizumab or immunotherapy alternative (avelumab, durvalumab)
- ❑ Clinical trial
- ❑ Other (please specify): ________

32. Has the patient progressed beyond second-line therapy?

- ❑ Yes

32.1. *[If selected, ask:]* **What third-line therapy was selected?**

- ❑ Reinduction with platinum-based chemotherapy
- ❑ Gemcitabine
- ❑ Paclitaxel

- ❑ Erdafitinib
- ❑ Enfortumab vedotin
- ❑ Pembrolizumab or immunotherapy alternative (avelumab, durvalumab)
- ❑ Clinical trial
- ❑ Other (please specify): ______________

❑ No

32.2. *[If selected, ask:]* **What third-line therapy are you planning in case of disease progression?**

- ❑ Reinduction with platinum-based chemotherapy
- ❑ Gemcitabine
- ❑ Paclitaxel
- ❑ Erdafitinib
- ❑ Enfortumab vedotin
- ❑ Pembrolizumab or immunotherapy alternative (avelumab, durvalumab)
- ❑ Clinical trial
- ❑ Too early to tell/ have not planned that far ahead
- ❑ Other (please specify): ________________

Reflection

33. What are your biggest challenges in managing patients on maintenance immunotherapy?

- ❑ Lack of experience with maintenance immunotherapy
- ❑ Managing treatment-emergent adverse events
- ❑ Lack of institutional resources (e.g., chemotherapy clinic already at capacity) to monitor and manage patients on maintenance immunotherapy
- ❑ Patient reluctance for further treatment (or frequency of treatment)
- ❑ Patient or resource factors limiting ability to start within 10 weeks
- ❑ Lack of access to maintenance therapy (i.e., no coverage)

34. What specific actions are you most motivated to make over the next 1-3 months?

- ❑ Review the latest guidelines
- ❑ Gain experience with maintenance immunotherapy
- ❑ Attend more educational programs to increase confidence in the management of these patients
- ❑ Learn more about the management of adverse events with maintenance immunotherapy
- ❑ Connect with peer(s) to discus what I have learned in this program
- ❑ This program has validated my current practice
- ❑ Other (please specify): _______

35. What challenges, if any, do you anticipate in implementing these plans for improvement? [Open text form]

Table S2. Discussion guide questions for focus groups

Background: Three focus groups lasting 90 minutes each were conducted between April 12 and 19, 2023. The three members of the scientific planning committee conducted the focus groups. They reviewed the aggregate findings from the chart review and gathered insights about the findings. Carefully crafted questions were added to guide the discussion around key elements of aUC management. The discussion questions are shown below.

1. **Do you see more *de novo* metastatic cases or more recurrences from earlier stages of disease?**
2. **What factors are involved in your decision to undergo (or not) a cystectomy?**
3. **Explain your creatinine clearance threshold when assessing eligibility for cisplatin.**
4. **What other factors do you consider when assessing a patient's eligibility for cisplatin?**
5. **Do you consider split-dosing cisplatin? If so, when?**
6. **How do you decide who should have molecular testing?**
7. **What challenges do you face with performing molecular testing in aUC?**
8. **What do your patients express as their top priority goals and how do they align with your priorities?**
9. **What factors drive you to choose between cisplatin and carboplatin?**
10. **How many cycles of chemotherapy do you typically treat for?**
11. **Are there circumstances where you would consider ddMVAC?**
12. **What is your toxicity threshold for discontinuing avelumab?**
13. **What could explain the high number of patients on active surveillance?**
14. **How do you choose between 2L treatment options?**
15. **What are your biggest challenges in managing patients on maintenance immunotherapy?**

Table S3. Additional clinical baseline characteristics

Characteristic	*n*	%
[Patients with disease progression from an earlier stage] **Primary tumor (T) stage when the patient was first diagnosed with localized disease (*n* = 69)**		
Ta, papillary tumor confined to the epithelium	1	1
T1, tumor invasion into the lamina propria	17	25
T2, tumor invasion into the muscularis propria	25	36
T3, tumor involvement of the perivesical fat	23	33
T4, tumor involvement of adjacent organs	3	4
[Patients with disease progression from an earlier stage] **Node (N) stage when the patient was first diagnosed with localized disease (*n* = 69)**		
N0, no regional lymph node metastasis	56	81
N1, metastasis in a single lymph node in true pelvis	8	12
N2, metastasis in multiple regional lymph nodes in true pelvis	5	7
N3, metastasis in common iliac lymph node(s)	0	0
**Steps involved in the initial evaluation of aUC (*n* = 146)**	146	100
History and physical examination	132	90
Estimate GFR to assess eligibility for cisplatin	94	64
Biopsy	79	54
Transurethral resection of bladder tumor (TURBT)	75	51
Cystoscopy (standard white light)	73	50
Urine cytology	34	23
Examination under anesthesia (bimanual)	12	8
Enhanced cystoscopy (e.g., fluorescence)	4	3
**Urinary tract imaging:**		
Computed tomography (CT) urogram	57	39
Renal ultrasound	13	9
Intravenous or retrograde pyelogram (IVP)	5	3
Magnetic resonance imaging (MRI) urogram	1	1
**Metastatic workup (imaging):**		
Chest imaging (CT scan or chest radiograph)	128	88
Bone scan (radionucleotide scan)	73	50
18F-fluorodeoxyglucose (FDG)-Positron emission tomography (PET)/CT	24	16
**Method used to assess kidney function**		
Measured creatinine clearance	1	1
Estimated creatinine clearance using Cockroft-Gault variant	110	75
Measured GFR	6	4
Estimated GFR using the MDRD equation	10	7
Estimated GFR using the CKD-EPI (2012) equation	1	1
Estimated GFR using the CKD-EPI (2021) equation	18	12

Table S4. Additional patient management patterns

Management component	*n*	%
**Priority treatment goals [1] (*n* = 146)**		
Improve survival	138	95
Delay progression-free survival	91	62
Improve response rates	91	62
Reduce burden of disease	46	32
Improve quality of life	25	17
Symptom management	24	16
Other [2]	1	1
**Reason for the selection of first-line therapy [3] (*n* = 146)**		
Most efficacious option for this patient	90	62
Tolerable	47	32
Eligible for cisplatin according to Galsky criteria	43	29
Favourable safety profile	42	29
NOT eligible for cisplatin according to Galsky criteria	38	26
Patient is fit	31	21
Patient is frail (e.g., multiple comorbidities, advanced age)	23	16
My "go-to" for most first-line patients	15	10
Associated with good adherence	11	8
Patient preference	6	4
Only therapy indicated for this patient	5	3
Other	1	1
**For patients currently on maintenance therapy or starting soon, who is involved in their care (aside from the oncologist)? (*n* = 67)**		
Clinic nurse	52	78
Pharmacist	48	72
Infusion nurse	28	42
Other [4]	4	6
**What is their main responsibility? [5]**		
Monitor patient for signs and symptoms of infusion-related reaction	20	30
Counsel after initial diagnosis	12	18
Monitor patient for treatment-emergent adverse events	11	16
Ongoing counsel during treatment	10	15
Manage supportive treatment(s)	7	10
Manage infusion-related reaction	6	9
Adjust concomitant medications	1	1
**For patients currently on maintenance therapy who experienced a 'difficult/time-consuming' AE, how was the safety event managed? (*n* = 5)**		
Managed with supportive treatment(s) (e.g., corticosteroids)	4	80
Withheld dose, no supportive treatment(s) required	1	20
Withheld dose, managed with supportive treatment(s)	1	20
Patient was referred to another specialist for further assessment	1	20
No intervention required	0	0
Permanent discontinuation of maintenance therapy	0	0
**For patients currently on maintenance therapy or under surveillance, what 2L therapy are you planning for this patient? (*n* = 81)**		
Pembrolizumab or immunotherapy alternative (avelumab, durvalumab)	34	42
Too early to tell/ have not planned that far ahead	24	30
Enfortumab vedotin	9	11
Reinduction with platinum-based chemotherapy	0	0

Gemcitabine	0	0
Paclitaxel	0	0
Erdafitinib	0	0
Clinical trial	0	0
Other	14	17
**For patients currently on 2L therapy, what 3L therapy are you planning for this patient upon disease progression? (*n* = 16)**		
Too early to tell/ have not planned that far ahead	5	31
Paclitaxel	2	13
Enfortumab vedotin	2	13
Erdafitinib	1	6
Pembrolizumab or immunotherapy alternative (avelumab, durvalumab)	1	6
Clinical trial	1	6
Reinduction with platinum-based chemotherapy	0	0
Gemcitabine	0	0
Other [6]	4	25

[1] Participants were asked to select their top 3 treatment goals from the options provided; % represents the percentage of participants who included this treatment goal in their top 3 priorities. [2] Another treatment goal was to delay symptomatic progression. [3] Participants were asked to select the top 3 reasons from the options provided. [4] Other providers involved in the care of patients currently on maintenance therapy are members of the oncology team and general practitioners in oncology. [5] Participants were intended to select all that apply, but a clerical error made is such that participants could only select one option. When queried about their roles and responsibilities during the focus group, participants indicated that the clinic nurse and pharmacist would be responsible for most of the roles that were listed. [6] Other 3L therapy options being considered were radiation (to residual lung metastases), and best supportive care (e.g., for patient refusing treatment).
